# 伴GATA2突变的骨髓增生异常肿瘤患者临床特征及预后分析

**DOI:** 10.3760/cma.j.cn121090-20260111-00019

**Published:** 2026-05

**Authors:** 静 唐, 玉娇 贾, 铁军 秦, 泽锋 徐, 士强 曲, 丽娟 潘, 清妍 高, 蒙 焦, 浩铨 徐, 泽飞 包, 琳琳 刘, 富慧 李, 颂扬 赵, 昕 王, 宁宁 柳, 文彬 安, 志坚 肖, 冰 李

**Affiliations:** 1 北京协和医学院，中国医学科学院血液病医院（中国医学科学院血液学研究所），血液与健康全国重点实验室，国家血液系统疾病临床医学研究中心，细胞生态海河实验室，天津 300020 State Key Laboratory of Experimental Hematology, National Clinical Research Center for Blood Diseases, Haihe Laboratory of Cell Ecosystem, Institute of Hematology & Blood Diseases Hospital, Chinese Academy of Medical Sciences & Peking Union Medical College, Tianjin 300020, China; 2 天津医学健康研究院，天津 301600 Tianjin Institutes of Health Science, Tianjin 301600, China

**Keywords:** 骨髓增生异常肿瘤, GATA2基因, 突变, 预后, Myelodysplastic neoplasms, GATA2 gene, Mutation, Prognosis

## Abstract

**目的:**

探讨伴GATA2突变的骨髓增生异常肿瘤（MDS）患者的临床特征及预后。

**方法:**

本研究为回顾性队列研究。收集2016年9月至2024年8月于中国医学科学院血液病医院确诊的1 365例MDS患者病例资料，分析伴GATA2突变MDS患者的临床特征、实验室特征及总生存（OS）情况。

**结果:**

①1 365例MDS患者中，69例（5.1％）检出致病或可能致病的GATA2突变，包括胚系/可能胚系突变14例（1.0％）、体细胞突变47例（3.4％）。胚系/可能胚系突变以功能缺失型突变为主［50.0％（7/14）］，体细胞突变以错义突变为主［76.8％（43/56）］。②GATA2胚系/可能胚系突变组中位确诊年龄小于体细胞突变组［34（13～68）岁对55（27～73）岁，*P*＝0.002］。共突变方面，胚系/可能胚系突变组STAG2突变检出率高于GATA2无突变组［42.9％（6/14）对3.2％（41/1 296），*P*<0.001］，体细胞突变组RUNX1（21.3％对9.7％）、BCOR（19.2％对5.9％）、SF3B1（31.9％对10.6％）突变检出率高于GATA2无突变组（*P*值均<0.05）。两组+8核型检出率均显著高于无突变组［胚系/可能胚系突变组：35.7％（5/14）对14.2％（164/1 158），*P*＝0.039；体细胞突变组：26.1％（12/46）对14.2％（164/1 158），*P*＝0.025］。③与胚系/可能胚系突变组相比，体细胞突变组中原始细胞增多（骨髓原始细胞≥5％或外周血原始细胞≥2％或存在Auer小体）患者比例更高［74.5％（35/47）对21.4％（3/14），*P*<0.001］。④在年龄<60岁的MDS患者中，GATA2体细胞突变组中位OS期短于GATA2无突变组［20（95％ *CI*：12～27）个月对未达到，*P*＝0.005］。多因素Cox分析提示，GATA2体细胞突变是年龄<60岁MDS患者独立于IPSS-M预后积分系统的不良预后因素（*HR*＝2.43，95％ *CI*：1.27～4.65，*P*＝0.007）。

**结论:**

GATA2胚系突变与体细胞突变的MDS患者具有不同的分子与临床特征。GATA2体细胞突变是年龄<60岁MDS患者独立的不良预后因素。

## Abstract (en)

**Objective:**

To investigate the clinical characteristics and prognosis of myelodysplastic neoplasms（MDS）patients with GATA2 mutations.

**Methods:**

This was a retrospective cohort study. Clinical data of 1 365 MDS patients who were diagnosed at the Institute of Hematology and Blood Diseases Hospital from September 2016 to August 2024 were collected. Retrospective analysis was conducted to evaluate the clinical features, laboratory characteristics, and overall survival（OS）of MDS patients with GATA2 mutations.

**Results:**

① Among the 1 365 MDS patients, 69（5.1％）carried pathogenic/likely pathogenic GATA2 mutations, including 14（1.0％）with germline/likely germline mutations and 47（3.4％）with somatic mutations. Null variants predominated in the germline/likely germline group［50.0％（7/14）］, whereas missense mutations were common in the somatic group［76.8％（43/56）］. ② The median age at diagnosis was significantly younger in the germline/likely germline group than in the somatic mutation group［34（13–68）years *vs* 55（27–73）years, *P*＝0.002］. The GATA2 germline/likely germline group had a significantly higher frequency of STAG2 mutations than the wild-type group［42.9％（6/14）*vs* 3.2％（41/1 296）, *P*<0.001］, whereas the somatic mutation group showed significantly higher frequencies of RUNX1（21.3％ *vs* 9.7％）, BCOR（19.2％ *vs* 5.9％）, and SF3B1（31.9％ *vs* 10.6％）mutations（all *P*<0.05）. Both GATA2 mutation groups had a significantly higher incidence of +8 karyotype than the wild-type group［germline/likely germline group: 35.7％（5/14）*vs* 14.2％（164/1 158）, *P*＝0.039; somatic mutation group: 26.1％（12/46）*vs* 14.2％（164/1 158）, *P*＝0.025］. ③ The proportion of patients with increased blasts（bone marrow blasts ≥5％ or peripheral blood blasts ≥2％ or presence of Auer rods）was significantly higher in the somatic mutation group than in the germline/likely germline group［74.5％（35/47）*vs* 21.4％（3/14）, *P*<0.001］. ④ Among the patients with MDS aged <60 years, the somatic GATA2 mutation group had a significantly shorter median OS than the wild-type group［20（95％ *CI*: 12–27）months vs not reached, *P*＝0.005］. Multivariate analysis revealed that GATA2 somatic mutation was an independent poor prognostic factor beyond the IPSS-M scoring system in patients with MDS aged <60 years（*HR*＝2.43, 95％ *CI*: 1.27–4.65, *P*＝0.007）.

**Conclusion:**

Patients with germline and somatic GATA2 mutations exhibit distinct molecular and clinical profiles. GATA2 somatic mutation is an independent poor prognostic factor in patients with MDS aged <60 years.

骨髓增生异常肿瘤（MDS）是一组具有高度异质性的起源于造血干细胞的髓系肿瘤性疾病，特征为髓系细胞发育异常、外周血细胞减少及高风险向急性髓系白血病（AML）转化[1]。2016年世界卫生组织（WHO）分型诊断标准首次提出将胚系易感髓系肿瘤（myeloid neoplasms associated with germline predisposition）列为独立的诊断分类[2]，GATA2缺陷综合征（G2DS）是其代表性疾病，通常起病于儿童或青少年期，表现为免疫缺陷、反复感染，高风险进展为MDS或AML并伴有特征性的细胞遗传学及分子遗传学[3]–[5]。体细胞GATA2突变亦可见于1％～4％的髓系肿瘤患者中[6]–[8]。目前国内关于伴GATA2突变的MDS患者临床队列研究较少，本文回顾性描述并分析伴有胚系或体细胞GATA2突变的MDS患者的临床特征、遗传学特征及预后。

## 病例与方法

1. 病例资料：本研究为回顾性队列研究，纳入2016年9月至2024年8月于中国医学科学院血液病医院确诊的具有完整病例资料的1 365例初诊原发性MDS患者，纳入标准：①符合WHO 2016年诊断分型标准；②完善血液肿瘤基因靶向二代测序（NGS）且不伴除GATA2基因突变（致病或可能致病性突变）外的致病或可能致病的先天性骨髓衰竭综合征相关基因突变。对所有纳入患者按照WHO 2022年标准[1]进行重新诊断分型。此外，为了后续进行总生存（OS）比较，将患者进一步分为2个亚组即原始细胞增多（骨髓原始细胞≥5％或外周血原始细胞≥2％或存在Auer小体）组和低原始细胞（骨髓原始细胞<5％且外周血原始细胞<2％且不存在Auer小体）组。采用修订版国际预后积分系统（IPSS-R）[9]、含分子遗传学指标的国际预后积分系统（IPSS-M）[10]对患者进行预后危险度分组。本研究经中国医学科学院血液病医院伦理委员会审批（批件号：IIT2021029-EC-1），并豁免患者知情同意。

2. NGS检测基因突变：分离患者骨髓单个核细胞，提取基因组DNA，经酶切打断片段化后，在两端连接测序接头（包含样本索引、通用PCR引物、流动槽结合序列及测序引物结合位点）。采用生物素标记的探针进行靶向区域杂交捕获，并用链霉亲和素磁珠富集目标文库；经扩增及文库混合后，使用Illumina NovaSeq 6000测序平台进行靶向测序，平均测序深度≥1 000×。下机数据进行生物信息学处理，首先根据样本索引拆分获得每个样本的FASTQ文件；然后将序列与GRCh37/hg19参考基因组对比，同时对原始比对数据进行去重处理；最后检测出目标区域内的单核苷酸变异（SNV）和小片段插入缺失（Indel）。变异经过质控过滤后，利用多个数据库（包括但不限于COSMIC、ClinVar、HGMD、ExAC和dbSNP）进行注释和初步筛选。筛选后剩余的变异经人工审查，并依据分子病理学协会（AMP）、美国临床肿瘤学会（ASCO）和美国病理学家协会（CAP）共同制定和发布的体细胞变异解读与报告指南[11]进行致病性分类。

3. GATA2胚系/可能胚系或体细胞突变判定：对于69例伴有GATA2致病或可能致病突变的MDS患者，通过口腔上皮细胞验证、治疗前后GATA2等位基因突变频率（VAF）比较并结合既往文献报道[12]–[16]对其进行判定，标准如下：①胚系/可能胚系突变（14例，20.3％）：初诊时口腔上皮细胞证实的胚系突变（6例，8.7％）；或经去甲基化治疗或联合化疗后评估疗效为完全缓解（CR）或骨髓完全缓解（mCR）且治疗前后VAF值均≥40％（1例，1.4％）；或无论治疗与否，伴或不伴GATA2突变相关病史或家族史，既往文献报道为胚系突变且VAF值≥40％（7例，10.1％）。②体细胞突变（47例，68.1％）：复测GATA2突变消失或复现或新发（14例，20.3％）；或仅有初诊测序且VAF值<40％（33例，47.8％）。③突变不能判定：不满足以上标准（8例，11.6％）。

4. GATA2突变类型分组：①功能缺失型突变（null variants）：定义为移码突变（frameshift mutation）、无义突变（nonsense mutation）、剪接位点突变（splice-site mutation）、大片段缺失（large deletion）和全基因缺失（whole-gene deletion）[13],[17]；②错义突变：错义突变（missense mutation）和框内突变（in-frame mutation）产生类似的突变效应[13]，后续分析时将两者统一划分为错义突变。

5. 年龄分组：为分析不同年龄段患者的伴随突变特征及GATA2突变类型差异，本研究将患者按年龄分为3组：≤39岁组、40～60岁组、>60岁组。分组依据如下：①GATA2胚系突变相关的MDS好发于儿童及年轻成人，既往报道其中位诊断年龄为12岁至34岁不等[5],[12]，多数患者在40岁之前确诊MDS或进展为AML[15]。美国国家综合癌症网络（NCCN）定义初诊年龄为15～39岁的患者为青少年和年轻成人[18]，与GATA2突变相关MDS的诊断年龄特征相符，本文以39岁作为青年患者的年龄界值。②参考《中国老年骨髓增生异常性肿瘤诊断和治疗专家共识（2024版）》[19]，以60岁作为老年患者的年龄界值。

6. 染色体核型分析：短期培养法常规制备染色体标本，采用R显带法分析核型，根据人类细胞遗传学国际命名体制（ISCN2016）描述染色体核型，并参照IPSS-R对染色体核型进行预后分组。

7. 随访：随访截止日期为2025年11月2日，随访资料通过查阅住院病历和门诊病历以及电话随访等方式收集。中位随访时间为17（1～168）个月，共63例（4.6％）患者失访。1 365例患者中，279例（20.4％）接受异基因造血干细胞移植（allo-HSCT）；69例伴有GATA2致病或可能致病突变的MDS患者中，16例（23.1％）接受allo-HSCT。总生存（OS）期定义为自确诊至任何原因死亡、末次随访日期或造血干细胞移植的时间。

8. 统计学处理：应用SPSS 29.0和GraphPad Prism 10.0进行统计分析和绘图，计量资料以*M*（范围）描述，组间比较采用Mann-Whitney *U*检验。计数资料以例数（构成比）表示，率的比较采用卡方检验或Fisher精确概率法。采用Kaplan-Meier法绘制生存曲线，Cox比例风险回归模型进行预后因素分析。双侧*P*<0.05认为差异具有统计学意义。

## 结果

1. GATA2基因突变频率和突变位点分布：本研究共纳入1 365例MDS患者，其中69例（5.1％）患者检出55种共计79个致病或可能致病的GATA2突变，包括胚系/可能胚系突变14例（1.0％）、体细胞突变47例（3.4％）、突变来源不能判定8例（0.6％）。79个致病或可能致病的GATA2突变的中位VAF值为22.3％（0.8％～56.2％）。突变类型以错义突变最常见（41个，51.9％），其次依次是移码突变（18个，22.8％）、框内突变（14个，17.7％）、无义突变（4个，5.1％），4号内含子突变和剪接位点突变各1个。突变位点主要位于第2个锌指结构域（ZF2区）（40个，50.6％）和第1个锌指结构域（ZF1区）（20个，25.3％）。ZF1区和ZF2区突变类型相似，非ZF区功能缺失型突变比例显著高于ZF1区［88.2％（15/17）对20.0％（5/20），*P*<0.001］和ZF2区［88.2％（15/17）对2.5％（1/40），*P*<0.001］。常见的GATA2突变位点为p.L359V（7个，8.9％）、p.A372T（6个，7.6％）和p.K390del（4个，5.0％）（图1）。

**图1:**
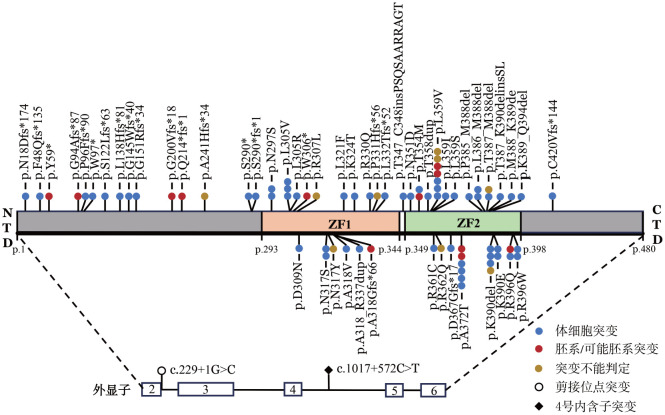
骨髓增生异常肿瘤患者GATA2基因突变位点分布模式图 **注** ZF1：第1个锌指结构域；ZF2：第2个锌指结构域；NTD：N端结构域；CTD：C端结构域

14例胚系/可能胚系突变患者的GATA2基因的中位VAF值为48.1％（41.1％～54.1％），47例体细胞突变患者的中位VAF值为8.3％（0.8％～44.1％）。胚系/可能胚系突变以功能缺失型突变为主［50.0％（7/14）］，体细胞突变以错义突变为主［76.8％（43/56）］，两组突变均最常发生于ZF2区（图1）。

2. GATA2突变患者的共突变和细胞遗传学特征：14例GATA2胚系/可能胚系突变和47例体细胞突变患者常见的基因共突变情况如附图1（**扫描本文首页二维码查阅**）所示。与1 296例无GATA2突变患者相比，GATA2胚系/可能胚系突变患者STAG2［42.9％（6/14）对3.2％（41/1 296），*P*<0.001］、JAK2［14.3％（2/14）对1.5％（19/1 296），*P*＝0.019］、FAT1［14.3％（2/14）对1.3％（17/1 296），*P*＝0.016］、CALR［7.1％（1/14）对0.2％（2/1 296），*P*＝0.031］突变检出率更高；而GATA2体细胞突变患者RUNX1［21.3％（10/47）对9.7％（126/1 296），*P*＝0.021］、BCOR［19.1％（9/47）对5.9％（76/1 296），*P*<0.001］、SF3B1［31.9％（15/47）对10.6％（137/1 296），*P*<0.001］、EZH2［14.9％（7/47）对4.5％（58/1 296），*P*＝0.006］、ETV6［8.5％（4/47）对2.5％（32/1 296），*P*＝0.034］突变检出率更高。

与GATA2错义突变/框内突变相比，GATA2功能缺失型突变倾向于与STAG2突变共同发生［37.5％（6/16）对6.1％（3/49），*P*＝0.005］。与ZF2区突变患者相比，ZF1区突变患者SF3B1突变发生率更高［47.4％（9/19）对11.8％（4/34），*P*＝0.007］，ZF2区突变患者ASXL1突变发生率高于ZF1区［35.3％（12/34）对5.3％（1/19），*P*＝0.019］，与非ZF区相比差异均无统计学意义（*P*值均>0.05）。

在细胞遗传学方面，无论是GATA2胚系/可能胚系突变［35.7％（5/14）对14.2％（164/1 158），*P*＝0.039］还是体细胞突变患者［26.1％（12/46）对14.2％（164/1 158），*P*＝0.025］，+8检出率均较无GATA2突变患者更高。涉及7号染色体异常、复杂核型等异常核型检出率，三组间差异均无统计学意义（*P*值均>0.05，**扫描本文首页二维码查阅附表1**）。

GATA2胚系/可能胚系突变患者中，错义突变发生率随年龄增长而升高［≤39岁组对40～60岁组对>60岁组：11.1％（1/9）对66.7％（2/3）对100％（2/2），*P*＝0.025］，功能缺失型突变则呈下降趋势［66.7％（6/9）对33.3％（1/3）对0（0/2），*P*＝0.336］（**扫描本文首页二维码查阅附图2A**）。GATA2体细胞突变患者中，+8核型检出率随年龄递减［42.9％（3/7）对27.3％（6/22）对17.6％（3/17），*P*＝0.413］，而RUNX1突变率随年龄呈递增趋势［0（0/7）对17.4％（4/23）对35.3％（6/17），*P*＝0.152］（**扫描本文首页二维码查阅附图2B**）。在共突变和细胞遗传学方面，胚系/可能胚系突变组中STAG2突变［55.6％（5/9）对33.3％（1/3）对0（0/2），*P*＝0.587］、+8核型［44.4％（5/9）对33.3％（1/3）对0（0/2），*P*＝0.751］及7号染色体异常［11.1％（1/9）对33.3％（1/3）对0（0/2），*P*＝0.614］在年轻患者中检出率更高，但差异均无统计学意义。而GATA2无突变组中，STAG2突变率随年龄增长显著升高［0.9％（2/227）对2.2％（13/585）对5.4％（26/484），*P*＝0.001］（**扫描本文首页二维码查阅附图2C**）。

3. GATA2突变患者的临床特征：GATA2突变与无突变患者的临床特征比较详见表1。胚系/可能胚系GATA2突变患者的起病和确诊中位年龄分别为26（6～67）岁和34（13～68）岁，均低于GATA2无突变的患者（*P*值均<0.001）。在胚系/可能胚系突变患者中，伴有STAG2突变患者起病至诊断的时间间隔更长［11（3～19）年对1（0～10）年，*P*＝0.016］。对所有患者依据WHO（2022）标准重新进行分型诊断。体细胞突变组［74.5％（35例）］中原始细胞增多患者比例显著高于无突变组［41.5％（538例），*P*<0.001］和胚系/可能胚系突变组［21.4％（3例），*P*<0.001］（表1）。无论是按照IPSS-R还是IPSS-M预后积分系统评估，GATA2体细胞突变患者较高危组（IPSS-R高危/极高危或IPSS-M中高危/高危/极高危）比例较无突变组更高，差异有统计学意义（*P*＝0.012和*P*＝0.021），而GATA2胚系/可能胚系突变组与无突变组相比差异无统计学意义（*P*>0.05，表1）。

**表1:** GATA2突变组与无突变组骨髓增生异常肿瘤（MDS）患者临床特征比较［例（％）］

临床特征	GATA2无突变组（1 296例）	GATA2突变组		统计量1	*P*1值	统计量2	*P*2值	统计量3	*P*3值
		胚系/可能胚系突变（14例）	体细胞突变（47例）						
男性	836（64.5）	7（50.0）	26（55.3）	–	0.272	*χ*^2^＝1.66	0.197	*χ*^2^＝0.12	0.726
确诊年龄［岁，*M*（范围）］	56（14~84）	34（13~68）	55（27~73）	*z*＝3.41	<0.001	*z*＝0.04	0.968	*z*＝3.14	0.002
起病年龄［岁，*M*（范围）］	54（4~84）	26（6~67）	55（20~73）	*z*＝3.52	<0.001	*z*＝0.04	0.964	*z*＝3.13	0.002
WBC［×10^9^/L，*M*（范围）］	2.62（0.27~27.95）	2.44（0.77~6.49）	2.65（0.18~11.70）	*z*＝0.63	0.528	*z*＝0.09	0.931	*z*＝0.47	0.637
ANC［×10^9^/L，*M*（范围）］	1.11（0~17.37）	1.16（0.24~3.82）	1.19（0.02~8.36）	*z*＝0.11	0.906	*z*＝0.51	0.604	*z*＝0.35	0.725
HGB［g/L，*M*（范围）］	79（19~158）	80（41~150）	78（46~145）	*z*＝0.26	0.790	*z*＝1.20	0.230	*z*＝0.93	0.350
PLT［×10^9^/L，*M*（范围）］	63（1~1 232）	144（8~286）	68（7~332）	*z*＝1.28	0.165	*z*＝0.57	0.563	*z*＝0.97	0.328
MCV［fl，*M*（范围）］	100.5（0.2~137.7）	101.6（85.0~116.8）	101.1（66.7~128.9）	*z*＝0.01	0.995	*z*＝0.35	0.720	*z*＝0.22	0.820
淋巴细胞绝对值［×10^9^/L，*M*（范围）］	1.11（0~4.23）	0.66（0.06~1.82）	1.01（0.14~3.30）	*z*＝1.92	0.054	*z*＝1.66	0.095	*z*＝0.98	0.327
单核细胞绝对值［×10^9^/L，*M*（范围）］	0.20（0~8.15）	0.04（0~0.90）	0.17（0.02~2.36）	*z*＝4.06	<0.001	*z*＝0.94	0.343	*z*＝3.27	0.001
WHO分型（2022）				–	0.272	–	<0.001	–	<0.001
MDS-5q−	20（1.5）	0（0）	0（0）						
MDS-SF3B1	115（8.9）	0（0）	3（6.4）						
MDS-biTP53	106（8.2）	0（0）	0（0）						
MDS-LB	454（35.0）	10（71.4）	8（17.0）						
MDS-h	110（8.5）	1（7.1）	1（2.1）						
MDS-IB	389（30.0）	2（14.3）	27（57.4）						
MDS-f	53（4.1）	0（0）	8（17.0）						
CCUS	10（0.8）	0（0）	0（0）						
AML	38（2.9）	1（7.1）	0（0）						
骨髓纤维化2~3级^a^	172（13.5）	1（7.1）	13（29.5）	–	0.708	*χ*^2^＝9.12	0.003	–	0.151
IPSS-R预后分组^b^				*χ*^2^＝0.45	0.831	*χ*^2^＝6.36	0.012	*χ*^2^＝1.08	0.297
极低/低/中危	690（59.9）	8（57.1）	19（41.3）						
高危/极高危	461（40.1）	6（42.9）	27（58.7）						
IPSS-M预后分组^c^				–	0.553	*χ*^2^＝5.34	0.021	–	0.698
极低/低/中低危	415（36.2）	3（25.0）	9（19.6）						
中高危/高危/极高危	731（63.8）	9（75.0）	37（80.4）						

**注** ANC：中性粒细胞绝对计数；MCV：平均红细胞体积；MDS-5q−：MDS伴孤立5q缺失；MDS-SF3B1：MDS伴SF3B1突变；MDS-biTP53：MDS伴TP53双等位基因改变；MDS-LB：MDS伴低原始细胞；MDS-h：低增生性MDS；MDS-IB：MDS伴原始细胞增多；MDS-f：MDS伴纤维化；CCUS：意义未明的克隆性血细胞减少症；AML：急性髓系白血病；IPSS-R：修订版国际预后积分系统；IPSS-M：含分子遗传学指标的国际预后积分系统；*P*1：无突变组对胚系/可能胚系突变组；*P*2：无突变组对体细胞突变组；*P*3：胚系/可能胚系突变组对体细胞突变组；^a^GATA2无突变组1 277例，GATA2胚系/可能胚系突变组14例，GATA2体细胞突变组47例；^b^GATA2无突变组共1 151例，138例患者无可分析的染色体核型、7例患者缺失中性粒细胞绝对值数据未进行IPSS-R预后分组，GATA2胚系/可能胚系突变组14例，GATA2体细胞突变组46例，1例患者无可分析的染色体核型未进行IPSS-R预后分组；^c^GATA2无突变组1 146例，138例患者无可分析的染色体核型、7例患者缺失中性粒细胞绝对值数据、5例患者年龄<18岁未进行IPSS-M预后分组，GATA2胚系/可能胚系突变组12例，2例患者年龄<18岁未进行IPSS-M预后分组，GATA2体细胞突变组46例，1例患者无可分析的染色体核型未进行IPSS-M预后分组；–：无统计值

4. GATA2突变患者预后：GATA2无突变、体细胞突变及胚系/可能胚系突变患者中位OS期分别为51（95％ *CI*：42～59）个月、24（95％ *CI*：16～31）个月和未达到，组间差异无统计学意义（*P*>0.05，**扫描本文首页二维码查阅附图3A**）。亚组分析显示，无论是伴原始细胞增多还是伴低原始细胞，三组间生存差异均无统计学意义（*P*值均>0.05，**扫描本文首页二维码查阅附图3B、C**）。年龄<60岁患者中，GATA2无突变、GATA2体细胞突变和GATA2胚系/可能胚系突变患者中位OS期分别为未达到、20（95％ *CI*：12～27）个月和未达到（图2），差异具有统计学意义（*P*＝0.017）。年龄≥60岁的亚组中，组间生存差异无统计学意义（*P*>0.05，**扫描本文首页二维码查阅附图3D**）。

**图2:**
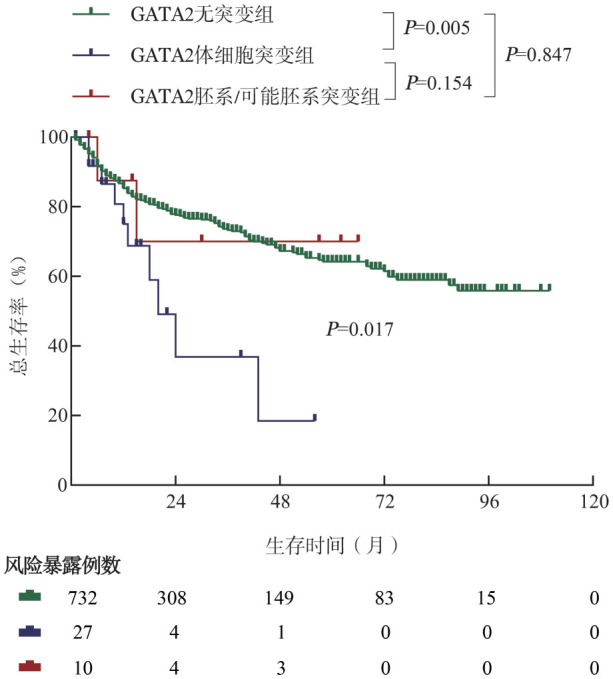
年龄<60岁亚组不同GATA2突变状态的骨髓增生异常肿瘤患者的总生存比较

在年龄<60岁患者中，对IPSS-M预后分组及GATA2突变状态进行Cox单因素回归分析，结果显示IPSS-M中高危/高危/极高危（*HR*＝3.11，95％ *CI*：2.16～4.49，*P*<0.001）、GATA2体细胞突变（*HR*＝2.73，95％ *CI*：1.44～5.20，*P*＝0.002）均与较差的OS相关。将上述变量纳入Cox多因素回归分析，结果显示GATA2体细胞突变（*HR*＝2.43，95％ *CI*：1.27～4.65，*P*＝0.007）是独立于IPSS-M预后积分系统的不良预后因素。

## 讨论

人类GATA2基因位于3q21.3，其编码的蛋白由480个氨基酸残基组成，主要参与维持造血干细胞的自我更新、增殖和分化，以及调控髓系和淋巴细胞分化发育。GATA2突变导致的功能缺失与免疫缺陷和髓系肿瘤遗传易感性有关[3]。本研究中，以成人患者为主的MDS队列中GATA2突变率为5.1％，明显低于儿童及青少年MDS患者中7.0％～8.5％的突变率[5],[20]。在成人MDS中，既往研究发现GATA2胚系突变发生率为0.5％～1.2％[21]–[22]，GATA2体细胞突变发生率为1％～4％[6]–[8]。本研究中，胚系/可能胚系突变率为1.0％，体细胞突变率为3.4％，与文献报道结果相似。

本研究中，50％GATA2胚系/可能胚系突变的成人MDS患者为功能缺失型突变，这与既往文献报道的伴GATA2胚系突变的儿童MDS以GATA2功能缺失型突变为主（52.8％）的结果基本一致[23]。GATA2体细胞突变最常见的突变类型为错义突变和框内突变，错义突变在ZF1和ZF2区均有分布，框内突变多集中在ZF2区，功能缺失型突变散在分布于非ZF区[24]–[25]，本研究中体细胞GATA2突变同样以错义突变为主（76.8％），突变分布区域与文献报道基本一致。

GATA2胚系突变的患者进一步获得体细胞突变和（或）细胞遗传学异常与其向髓系肿瘤进展相关[14]。这类患者常发生ASXL1、SETBP1、STAG2等基因突变，以及−7/7q−、+8、der（1；7）等细胞遗传学异常。一项针对伴有GATA2胚系突变的180例儿童和38例成人MDS患者的研究显示，25.9％的患者伴有SETBP1突变，76.5％的患者伴有−7[23]。SETBP1突变和−7是儿童MDS患者最常见的获得性基因突变和细胞遗传学异常，并且与更早发生MDS转化有关，而STAG2突变（突变频率14.7％）和+8（突变频率53.3％）也是常见的获得性基因突变和细胞遗传学异常，与较晚发生MDS转化有关。本研究中，GATA2胚系/可能胚系突变患者的STAG2突变比例高达42.9％，+8比例高达35.7％，但−7/7q−比例仅为21.4％，且未检出SETBP1突变。本研究进一步提示，不同的获得性基因突变和细胞遗传学异常驱动GATA2胚系突变患者发生MDS转化的动力学具有显著差异。此外，本研究发现无论是伴有胚系还是体细胞GATA2突变，RUNX1突变频率均较高，分别为21.4％和21.3％。尽管STAG2和造血转录因子RUNX1的相互作用被认为可导致MDS发生[26]，但本研究中STAG2与RUNX1突变很少同时发生。合并STAG2突变可以延缓伴有GATA2胚系突变的儿童患者发生MDS的现象可以解释为什么成人GATA2胚系突变患者中合并STAG2突变较为常见[23]，但其具体分子机制仍未明确，可能与儿童SAMD9/SAMD9L胚系突变伴−7核型异常的体细胞挽救（somatic rescue）类似[27]。RUNX1和GATA2是造血过程中关键的转录因子，其在调控造血干细胞分化、特别是髓系谱系的发育中存在紧密且复杂的相互关系[28]，因此在GATA2胚系突变合并获得性STAG2突变背景下，两者可能存在互斥现象。

本研究发现MDS伴原始细胞增多患者中，GATA2胚系/可能胚系突变患者较体细胞突变患者预后更差。对GATA2缺陷患者进行长期随访的研究表明，GATA2缺陷的MDS或AML患者对化疗反应差、发生严重感染及疾病进展的风险高[15],[29]。本研究中2例伴原始细胞增多的GATA2胚系/可能胚系突变患者，在接受地西他滨去甲基化治疗后均未获得疗效反应，并最终死于重症感染，其中1例在短期内进展为急性白血病，该亚组患者的不良预后主要与去甲基化治疗疗效差和感染风险高相关。在年龄<60岁的患者中，GATA2体细胞突变是独立于IPSS-M预后积分系统的不良预后因素。这提示GATA2体细胞突变可能在年轻MDS患者中定义了一个独特的疾病亚型。

综上所述，本研究系统描述了我中心伴有GATA2突变的成人MDS患者的分子学、细胞遗传学、临床特征和预后。本研究尚存在以下不足：①经过口腔黏膜上皮细胞、趾甲/指甲或毛囊验证的GATA2胚系突变患者比例较低，可能影响亚组分析的准确性；②由于伴有GATA2突变的MDS患者数量有限且随访时间较短，其对于预后的影响有待进一步多中心、前瞻性研究加以验证。

## Supplementary Material



## References

[1] Khoury JD, Solary E, Abla O (2022). The 5th edition of the World Health Organization classification of haematolymphoid tumours: myeloid and histiocytic/dendritic neoplasms[J]. Leukemia.

[2] Arber DA, Orazi A, Hasserjian R (2016). The 2016 revision to the World Health Organization classification of myeloid neoplasms and acute leukemia[J]. Blood.

[3] Liu YC, Eldomery MK, Maciaszek JL (2025). Inherited predispositions to myeloid neoplasms: pathogenesis and clinical implications[J]. Annu Rev Pathol.

[4] Calvo KR, Hickstein DD (2023). The spectrum of GATA2 deficiency syndrome[J]. Blood.

[5] Wlodarski MW, Hirabayashi S, Pastor V (2016). Prevalence, clinical characteristics, and prognosis of GATA2-related myelodysplastic syndromes in children and adolescents[J]. Blood.

[6] Lindsley RC, Mar BG, Mazzola E (2015). Acute myeloid leukemia ontogeny is defined by distinct somatic mutations[J]. Blood.

[7] Papaemmanuil E, Gerstung M, Bullinger L (2016). Genomic classification and prognosis in acute myeloid leukemia[J]. N Engl J Med.

[8] Papaemmanuil E, Gerstung M, Malcovati L (2013). Clinical and biological implications of driver mutations in myelodysplastic syndromes[J]. Blood.

[9] Greenberg PL, Tuechler H, Schanz J (2012). Revised international prognostic scoring system for myelodysplastic syndromes[J]. Blood.

[10] Bernard E, Tuechler H, Greenberg PL (2022). Molecular international prognostic scoring system for myelodysplastic syndromes[J]. NEJM Evid.

[11] Li MM, Datto M, Duncavage EJ (2017). Standards and guidelines for the interpretation and reporting of sequence variants in cancer: a joint consensus recommendation of the Association for Molecular Pathology, American Society of Clinical Oncology, and College of American Pathologists[J]. J Mol Diagn.

[12] Spinner MA, Sanchez LA, Hsu AP (2014). GATA2 deficiency: a protean disorder of hematopoiesis, lymphatics, and immunity[J]. Blood.

[13] Homan CC, Venugopal P, Arts P (2021). GATA2 deficiency syndrome: a decade of discovery[J]. Hum Mutat.

[14] West RR, Calvo KR, Embree LJ (2022). ASXL1 and STAG2 are common mutations in GATA2 deficiency patients with bone marrow disease and myelodysplastic syndrome[J]. Blood Adv.

[15] Donadieu J, Lamant M, Fieschi C (2018). Natural history of GATA2 deficiency in a survey of 79 French and Belgian patients[J]. Haematologica.

[16] Hahn CN, Chong CE, Carmichael CL (2011). Heritable GATA2 mutations associated with familial myelodysplastic syndrome and acute myeloid leukemia[J]. Nat Genet.

[17] Largeaud L, Collin M, Monselet N (2023). Somatic genetic alterations predict hematological progression in GATA2 deficiency[J]. Haematologica.

[18] National Comprehensive Cancer Network (NCCN) NCCN Guidelines for Patients: Adolescent and Young Adult Cancer[EB/OL].

[19] 中国老年医学学会血液学分会MDS专委会 (2024). 中国老年骨髓增生异常性肿瘤诊断和治疗专家共识(2024版)[J]. 诊断学理论与实践.

[20] 安 文彬, 刘 超, 万 扬 (2019). GATA2突变相关儿童原发性骨髓增生异常综合征临床及分子生物学特征[J]. 中华血液学杂志.

[21] Feurstein S, Trottier AM, Estrada-Merly N (2022). Germ line predisposition variants occur in myelodysplastic syndrome patients of all ages[J]. Blood.

[22] Sahoo SS, Kozyra EJ, Wlodarski MW (2020). Germline predisposition in myeloid neoplasms: Unique genetic and clinical features of GATA2 deficiency and SAMD9/SAMD9L syndromes[J]. Best Pract Res Clin Haematol.

[23] Kotmayer L, Kozyra EJ, Kang G (2025). Age-dependent phenotypic and molecular evolution of pediatric MDS arising from GATA2 deficiency[J]. Blood Cancer J.

[24] Nanaa A, Viswanatha D, Xie Z (2021). Clinical and biological characteristics and prognostic impact of somatic GATA2 mutations in myeloid malignancies: a single institution experience[J]. Blood Cancer J.

[25] Jabban Y, He R, Bessonen K (2025). Clinical characteristics and prognostic significance of co-mutated SETBP1/GATA2 myeloid neoplasms[J]. Leuk Res.

[26] Ochi Y, Kon A, Sakata T (2020). Combined cohesin-RUNX1 deficiency synergistically perturbs chromatin looping and causes myelodysplastic syndromes[J]. Cancer Discov.

[27] Sahoo SS, Pastor VB, Goodings C (2021). Clinical evolution, genetic landscape and trajectories of clonal hematopoiesis in SAMD9/SAMD9L syndromes[J]. Nat Med.

[28] Bresciani E, Carrington B, Yu K (2021). Redundant mechanisms driven independently by RUNX1 and GATA2 for hematopoietic development[J]. Blood Adv.

[29] Pasquet M, Bellanné-Chantelot C, Tavitian S (2013). High frequency of GATA2 mutations in patients with mild chronic neutropenia evolving to MonoMac syndrome, myelodysplasia, and acute myeloid leukemia[J]. Blood.

