# Supplementary figures and images for: Clinical characteristics and prognosis of myelodysplastic neoplasms with GATA2 mutations
Source: Zhonghua Xue Ye Xue Za Zhi. 2026 May;47(5):465–72. [Article in Chinese] doi: 10.3760/cma.j.cn121090-20260111-00019 (PMC13416548; doi:10.3760/cma.j.cn121090-20260111-00019)

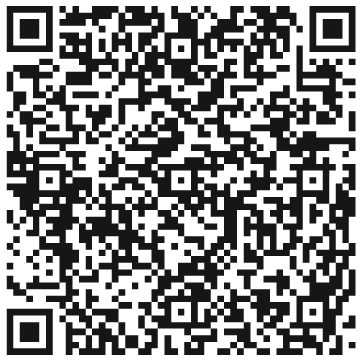

Supplement: Supplementary file 1 [file cjh-47-05-465-g003.tif]
